# Research progress in exercise-induced fatigue monitoring and injury early warning based on flexible sensing textiles and deep learning

**DOI:** 10.3389/fbioe.2026.1875539

**Published:** 2026-07-08

**Authors:** Dan Chen, Hao Chen, Jianqiang Guo

**Affiliations:** 1 School of Physical Education and Health, Shanghai Lixin University of Accounting and Finance, Shanghai, China; 2 School of Physical Education, Changzhou University, Changzhou, China

**Keywords:** deep learning, exercise-induced fatigue, flexible sensing textiles, injury early warning, sports biomechanics

## Abstract

Accurate identification of exercise induced fatigue and real time injury early warning is a core requirement for scientific training in competitive sports. Traditional laboratory based biomechanical monitoring is hindered by spatial constraints and limited ecological validity. The integration of flexible sensing textiles and deep learning has emerged as a disruptive solution. This paper reviews recent progress in flexible sensing textiles for athlete monitoring. First, the mechanical response characteristics of Frontier sensing technologies including self-powered triboelectric nanogenerators, piezoresistive or capacitive sensors, and liquid metals are analyzed for capturing microscopic biomechanical signals. Second, deep learning architectures such as CNN, Long Short-Term Memory, and Transformers are discussed for signal denoising, action phase segmentation, and fatigue feature mining. Crucially, the early warning logic based on the fatigue compensation injury causal chain is elaborated, covering real-time high-risk movement monitoring for acute injuries, cumulative load evaluation for overuse injuries, and digital twin driven individualized benchmarking. Finally, future challenges including signal robustness, washability, and multimodal data fusion are addressed. This review establishes a biomechanically informed theoretical framework for smart sports apparel, facilitating a paradigm shift toward closed loop intelligent prediction in injury prevention.

## 1 Introduction

In the landscape of modern high-performance athletic training, the accurate quantification of training load and fatigue status constitutes the core pathway for optimizing competitive performance and mitigating injury risk (Soligard et al., 2016). As intensity requirements in professional sports continue to escalate, the accumulation of exercise-induced fatigue has become a primary driver of both acute and chronic musculoskeletal injuries (Bahr and Krosshaug, 2005). According to an 18-year prospective study by Ekstrand et al. (2021) on elite European football clubs, despite continuous advancements in sports medicine, the incidence of fatigue-related muscle injuries has not shown a significant decline, remaining relatively constant. Conventional functional monitoring tools, such as the Session Rating of Perceived Exertion (RPE) or periodic physiological markers (e.g., blood lactate, heart rate variability), often suffer from inherent latency, failing to provide immediate kinematic and kinetic feedback within real-world competitive scenarios (Wu et al., 2024; Mateo-March et al., 2023).

Standard biomechanical monitoring traditionally relies on optoelectronic motion capture systems (e.g., Vicon) and three-dimensional force plates (Elliott et al., 2006). Although these instruments are regarded as the gold standard, their dependence on constrained laboratory environments severely decontextualizes the athlete’s true competitive state, leading to a profound lack of ecological validity in experimental results (Tsay et al., 2024). In the current digital era, wearable inertial measurement units (IMUs) based on micro-electromechanical systems (MEMS) have gained popularity for field-based monitoring. However, their rigid packaging and battery endurance bottlenecks often induce motion artifacts and may interfere with the athlete’s proprioception (Rong and Kuo, 2024; Basel et al., 2021). Consequently, there is an urgent demand in sports science for a novel monitoring medium that is unobtrusively integrated, long-lasting, self-powered, and capable of high-fidelity signal acquisition (Zhang W.-W. et al., 2025).

The emergence of Flexible Sensing Textiles (FSTs) provides a promising solution to many of the limitations associated with conventional wearable monitoring systems (Xu et al., 2025). By integrating advanced functional nanomaterials, such as carbon nanotubes, MXenes, and liquid metals, into textile fiber structures, smart fabrics can achieve seamless integration between sensing functions and wearable garments. These systems are capable of continuously monitoring biomechanical parameters, including joint angle variations, contact time, and plantar pressure distribution. Importantly, they can maintain user comfort through desirable characteristics such as breathability, flexibility, and washability (Hannigan et al., 2024; Wang Y. et al., 2025). In addition, the development of triboelectric nanogenerator (TENG) technology has enabled a new generation of self-powered sensing systems. By harvesting mechanical energy generated during human movement and converting it into electrical signals, TENG-based sensors can reduce dependence on external power supplies. This capability is particularly valuable for long-duration monitoring applications, such as marathon running and triathlon competitions (Feng et al., 2025; Wang and He, 2025).

Nevertheless, the raw signals generated by flexible textile sensors in field environments are typically accompanied by high non-linear noise and significant inter-individual variability, rendering traditional linear regression models inadequate for precise fatigue identification (Ballester et al., 2024). Recently, the introduction of Deep Learning (DL) algorithms has substantially enhanced the efficacy of sports data analytics (Jia et al., 2025). By utilizing Convolutional Neural Networks to automatically extract morphological fingerprints from motion waveforms and Long Short-Term Memory networks to process temporal dependencies in fatigue evolution, researchers can now accurately identify critical indicators such as cadence variability and gait asymmetry in fatigued states (Gao et al., 2023). This mapping from low-level physical signals to high-level competitive performance evaluation establishes the algorithmic foundation for constructing individualized injury risk early warning models (Xu, 2026).

**FIGURE 1:**
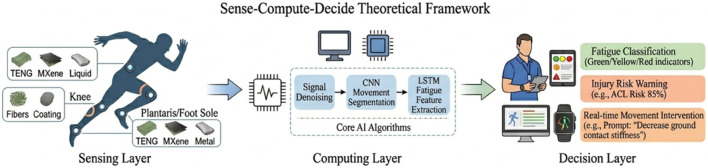
Architecture of the closed-loop system for fatigue monitoring and injury early warning in smart sports.

Despite rapid advancements, a comprehensive review that integrates the triad of “materials-algorithms-sport science logic” remains scarce. Existing literature predominantly focuses on isolated dimensions, either discussing the fabrication and optimization of flexible sensing materials or emphasizing deep learning applications in wearable signal processing; few studies have incorporated sensing technology, deep learning, and the biomechanical representation logic of fatigue into a unified analytical framework. Therefore, this paper aims to bridge this gap by systematically reviewing the research progress of flexible sensing textiles in athlete monitoring over recent years. It specifically examines the mechanical response characteristics of diverse sensing technologies, the application pathways of deep learning in fatigue recognition, and injury prevention strategies based on real-time kinetic data, ultimately providing a theoretical framework for the development of future smart sports apparel. To visualize the theoretical system established in this study, Figure 1 illustrates the end-to-end system architecture, spanning from bottom-layer flexible sensing to mid-layer deep learning computation and top-layer injury early warning decision-making.

To facilitate the synthesis of this highly interdisciplinary field, this review proposes four conceptual propositions (P1–P4) rather than empirically testable hypotheses. These propositions are intended to serve as an analytical framework for organizing, interpreting, and discussing the current body of evidence. Rather than representing statistically validated hypotheses, they provide a conceptual lens through which the relationships among flexible sensing textiles, deep learning, fatigue monitoring, and injury prevention can be understood.

P1: Flexible sensing textiles enable continuous acquisition of multidimensional biomechanical and physiological signals during athletic activities, providing an important foundation for real-time sports monitoring.
P2: Deep learning approaches can extract fatigue-related information from complex and noisy sensor signals and demonstrate considerable potential for improving fatigue recognition and assessment.
P3: The integration of wearable sensing data and intelligent analytical models facilitates injury risk stratification and early warning by identifying fatigue-related biomechanical adaptations that may be associated with elevated injury risk.
P4: Closed-loop systems integrating real-time sensing, intelligent analysis, and personalized feedback may improve the effectiveness of fatigue management and injury prevention strategies in sports settings.

### 1.1 Literature search strategy and study selection

To improve the transparency and reproducibility of this review, a structured literature search and study selection strategy was adopted. Given the interdisciplinary nature of flexible sensing textiles, deep learning, sports biomechanics, fatigue monitoring, and injury risk assessment, the present work was designed as a comprehensive narrative review supported by a structured literature search. While not a formal systematic review or meta-analysis, selected transparency principles recommended by the PRISMA framework were considered during literature identification and screening.

Relevant literature was retrieved from multiple academic databases, including Web of Science Core Collection, Scopus, PubMed, IEEE Xplore and Google Scholar. The search focused on studies published between 2015 and 2026. To capture recent developments in rapidly evolving areas such as wearable artificial intelligence, smart textiles, and digital twins, a limited number of highly relevant early-access publications, preprints, and patent-related studies were also considered when peer-reviewed evidence was limited.

Representative search terms included combinations of the following keywords: “flexible sensing textile”, “smart textile”, “electronic textile”, “wearable sensor”, “exercise-induced fatigue”, “fatigue monitoring”, “injury prevention”, “injury risk assessment”, “sports biomechanics”, “deep learning”, “machine learning”, “artificial intelligence”, “digital twin”, and “wearable intelligence”.

Studies were considered eligible if they: (1) focused on flexible sensing textiles or wearable sensing technologies; (2) involved fatigue monitoring, biomechanical assessment, injury risk evaluation, or intelligent sports applications; (3) incorporated machine learning, deep learning, or artificial intelligence techniques; and (4) provided sufficient methodological or technical information for analysis. Studies were excluded if they were duplicate records, editorials, conference abstracts without accessible full text, or articles unrelated to sports monitoring and injury prevention.

The retrieved literature was screened through a multi-stage evaluation of titles, abstracts, and full texts. Priority was given to peer-reviewed journal articles with clear methodological descriptions and experimental validation. However, considering the rapid pace of technological development in this field, selected preprints, patents, and emerging studies were included when they provided unique technical insights. To ensure academic rigor, a three-fold internal reliability assessment framework was applied to screen these non-peer-reviewed sources, evaluating: (1) methodological and fabrication transparency (e.g., explicit hardware parameters); (2) rigorous algorithmic configurations and standard validation protocols (e.g., explicit sliding-window configurations and quantitative benchmarks like RMSE and recall metrics); and (3) data/code transparency or subsequent peer recognition.

Following screening and relevance assessment, the selected studies were categorized according to sensing materials, sensing mechanisms, deep learning architectures, fatigue monitoring applications, injury risk assessment strategies, and intelligent intervention frameworks. This classification formed the foundation of the proposed materials–algorithms–sports biomechanics framework.

Nevertheless, because this review includes emerging technologies and a limited number of preprints and patent-related studies, inherent limitations regarding the lack of formal peer review for these specific sources persist. Some conclusions derived from these emerging works should be interpreted as indicative of current research trends and future directions rather than definitive evidence.

## 2 Classification of flexible sensing textiles and biomechanical response logic

Flexible sensing textiles (FSTs) serve as the foundational hardware for intelligent sports monitoring systems. Their primary function is to transduce complex biomechanical information—such as strain, stress, and acceleration—into quantifiable electrical signals (Ma et al., 2021). Unlike traditional laboratory-grade stationary sensors, real-world athletic scenarios require sensing media to possess exceptional flexibility, dynamic stability, and biocompatibility. Depending on the energy conversion mechanism and material response characteristics, current research focuses on self-powered triboelectric systems, high-performance piezoresistive/capacitive sensors, and novel fluid materials designed for extreme deformation.

### 2.1 Triboelectric nanogenerators (TENGs)

Based on the coupling of contact electrification and electrostatic induction, TENGs offer a battery-free, closed-loop solution for long-term monitoring in competitive sports (Wang Y. et al., 2025). In endurance events such as marathons and race walking, device weight and battery life are critical constraints for data integrity. TENG-based textiles directly convert mechanical energy from an athlete’s gait into high-signal-to-noise ratio (SNR) voltage signals, where the output characteristics exhibit a high degree of biomechanical correlation with exercise load (Li J. et al., 2025). According to recent studies, high-performance TENG fibers typically utilize conductive yarns with multi-level core-shell structures. These fibers enhance charge density by incorporating nanocomposites with significant electronegative/electropositive differences, such as carbon nanotube-doped silicone rubber or micro-structured fluoropolymers (Wang et al., 2024; Domingos et al., 2025). From the perspective of biomechanical representation logic, the peak voltage of TENG signals scales linearly with the angular velocity of limb swings, while the charge transfer corresponds to the displacement amplitude of muscle contractions. Research indicates that microsecond-level fluctuations in contact time and flight time captured by TENG textiles can sensitively reflect changes in gait stiffness induced by neuromuscular fatigue, providing a pristine physical fingerprint for fatigue identification (Aliyana et al., 2025; Johns et al., 2025).

### 2.2 Piezoresistive and capacitive sensing technologies

Piezoresistive and capacitive sensors are the predominant choices for analyzing high-frequency, high-impact technical movements, such as directional changes in football and explosive power in weightlifting (Yin and Sun, 2025). Piezoresistive textiles function by sensing changes in the contact resistance of internal conductive networks—typically composed of MXenes, graphene, or metallic nanowires—under pressure. Wang et al. (2023) proposed a strain sensor based on a hierarchical microcrack structure in MXene/AgNW composite films, achieving a high sensitivity (Gauge Factor ≈244) across a 0%–60% strain range and a response time of less than 30 m. This ultra-high sensitivity enables the detection of both large-scale movements and subtle human motions, including the potential to capture distal tremors, which is invaluable for precision monitoring in static sports like shooting and archery. Capacitive sensors, conversely, excel in kinetic monitoring due to their excellent linear response and minimal hysteresis (Meena et al., 2023). Recent innovations involving porous elastomer structures in the dielectric layer have significantly enhanced sensor sensitivity within high-pressure ranges (e.g., impact forces) (Zhang X. et al., 2025). In high-collision sports like rugby and ice hockey, capacitive sensing textiles provide stable estimations of Ground Reaction Force (GRF), enabling coaches to evaluate an athlete’s energy dissipation efficiency and buffering capacity under fatigued states (Middleton et al., 2025; Straub and Pott, 2025).

### 2.3 Liquid metals and electrospun textiles

For movements involving extreme limb rotation and stretching (strains often exceeding 200%) in sports such as gymnastics, diving, and throwing, traditional solid conductive layers are prone to crack propagation or signal failure (Hajalilou, 2025). Liquid Metals (LMs), such as EGaIn (Gallium-Indium alloy), have emerged as sensing saviors for extreme deformation scenarios due to their fluid-like mobility and metallic conductivity (Geng et al., 2025; Laperrousaz et al., 2025). A study published by Duan et al. (2025) in 《Nature Communications》 demonstrated all-textile integrated liquid metal fibers that maintain highly consistent resistance even after 5,000 cycles of stretching, ensuring zero-drift signaling during complex athletic maneuvers. To mitigate sweat interference, Wu et al. (2026) utilized electrospinning to fabricate spindle-structured directional sweat-wicking nanotextiles, achieving an ultra-fast unidirectional transport rate of 4.00 mL min^-1^·cm^-2^ (approximately 1,200 times the human perspiration rate). This allowed for the simultaneous acquisition of ECG and sweat glucose signals under exercise conditions. Similarly, Li et al. (2026) constructed fiber-based vertical organic electrochemical transistors (vOECTs) using an all-electrospinning strategy, which stably recorded ECG signals despite perspiration and skin deformation, maintaining performance over 10,000 deformation cycles. These textile-based sensors effectively resolve signal interference caused by interface sweat accumulation, facilitating all-weather performance monitoring in winter sports and high-humidity training environments.

### 2.4 Biomechanical sensor placement and decoupling logic

The efficacy of flexible sensing textiles depends not only on the sensing mechanism but also on their anatomical layout within the garment. Current trends favor distributed sensing based on anatomical landmarks, such as embedding sensing units with varying sensitivities at the anterior cruciate ligament (ACL), Achilles tendon, and rectus abdominis (Liu et al., 2025). This distributed logic allows for the decoupling of complex whole-body movements into independent biomechanical degrees of freedom. Through preliminary hardware-level filtering and structuralization, this approach significantly alleviates the burden on deep learning algorithms to process motion artifacts, thereby providing the physical assurance necessary for real-time functional monitoring.

### 2.5 Adaptation matrix between sensing technologies and sports monitoring requirements

In conclusion, the physical response characteristics of flexible sensing textiles determine their efficacy in capturing biomechanical signals of varying natures. The heterogeneity of exercise-induced fatigue necessitates a differential hardware matching logic, as summarized in Table 1.

**TABLE 1:** Hardware Adaptation Matrix between flexible sensing technologies and sports fatigue monitoring requirements.

Fatigue category	Core biomechanical fingerprints	Recommended sensing technology path	Key response characteristics	Representative literature
Peripheral Muscle Fatigue	local micro-tremors,force frequency deviation	high-sensitivity Piezoresistive (e.g., MXene-coated textiles)	ultra-high sensitivity (GF > 200), response time <30 m	Wang et al. (2023), Middleton et al. (2025)
Central Gait Fatigue	cadence drift, contact time variability	self-powered Triboelectric (TENG fibers/insoles)	high temporal resolution, self-powered, long-term cyclic stability	Aliyana et al. (2025), Johns et al. (2025), Wang Y. et al. (2025)
Compensatory Technical Fatigue	decreased joint ROM, kinetic chain instability	liquid metal/strain fibers (integrated into braces)	ultra-large stretchable range (>200%), low mechanical hysteresis	Duan et al. (2025), Li et al. (2026)
All-weather Field Monitoring	interference under sweat penetration (ECG/mechanical)	electrospun directional sweat-wicking textiles	ultra-fast unidirectional transport, stable against humidity interference	Wu et al. (2026), Li et al. (2026)

First, the physiological hallmarks of peripheral muscle fatigue primarily manifest as micro-tremors of local muscle fibers. Capturing these high-frequency, subtle disturbances require sensors to possess an exceptionally high Gauge Factor within low-pressure regimes. Consequently, high sensitivity piezoresistive sensors based on MXenes or graphene emerge as the optimal solution for this scenario. In contrast, central gait fatigue is characterized more by non-linear drifts in macro-kinetic rhythms, such as prolonged contact times and fluctuations in cadence. This mode of long-duration monitoring places stringent demands on the temporal resolution and cyclic stability of the device. Self-powered TENG technology, with its battery-free operational advantage and high-signal-to-noise ratio time-stamp response, exhibits superior ecological validity for monitoring gait asymmetry. Furthermore, as fatigue accumulates and triggers kinetic chain compensation and technical deformation, it is often accompanied by significant alterations in the joint Range of Motion. In such instances, large-range sensing materials, such as liquid metals, ensure that the sensors maintain signal fidelity without non-linear distortion under extreme deformation (strains often exceeding 200%), thereby providing robust data support for the precise identification of erroneous compensatory movements induced by fatigue.

It is worth noting that this hardware adaptation matrix serves as the first stage (the physical data-acquisition layer) of the entire monitoring pipeline. The physical characteristics compiled here (e.g., temporal resolution of TENG, topological distribution of liquid metal arrays) directly define the mathematical structure of the output data, which subsequently dictates the selection of downstream deep learning architectures discussed in Section 3.4.

## 3 Deep learning-driven paradigms for motion signal processing and feature analytics

The raw electrical outputs generated by flexible sensing textiles (FSTs) in real-world athletic environments are characterized by profound non-stationarity and stochastic interference. Due to the high-frequency oscillation of limbs and electrolyte effects at the skin-textile interface (e.g., sweat penetration), signals are frequently coupled with complex motion artifacts and baseline drifts (Ji et al., 2024). Consequently, constructing a deep learning architecture capable of transitioning from low-level physical denoising to high-level logical extraction is a mathematical prerequisite for the precise identification of fatigue.

### 3.1 Signal denoising and preprocessing

The primary challenge for FSTs lies in the complex force-electricity-biology coupling interference. Motion artifacts predominantly arise from relative displacement between the textile and the skin, as well as parasitic charges generated by garment folds (Michael and Howard, 2017). Furthermore, transient fluctuations in interface conductivity caused by profuse perspiration often induce severe baseline drifts. Current academic frontiers have shifted away from traditional linear Butterworth filtering toward adaptive denoising schemes based on Autoencoders (AE) or Generative Adversarial Networks (GAN) (Peng and Li, 2025; Guo et al., 2026). Lim et al. (2026) employed a CNN encoder-decoder architecture (CNNAED) to suppress motion artifacts in wearable ECGs, reducing heart rate estimation error by 29.3%. Guo et al. (2025) further proposed a Spatio-Temporal Interaction Feature Enhanced Autoencoder (ST-HCEAE) model, which captures global and local spatio-temporal correlations between process variables and utilizes hierarchical complementary enhancement modules to resolve the issues of low-level feature susceptibility to noise and the layer-wise loss of high-level information. By reconstructing pristine biomechanical feature maps within a latent space, AI algorithms can effectively decouple physical noise from motion-relevant signals in the time-frequency domain. Additionally, the fusion of Empirical Mode Decomposition (EMD) and Deep Residual Networks (ResNet) has demonstrated exceptional robustness in processing transient signals during high-frequency collisions (e.g., football tackling or rapid directional changes). Hu et al. (2025) proposed a joint WPD-EMD denoising method combined with an improved ResNet architecture, achieving a 93.83% overall recognition rate for transient signals and up to 99.69% for specific impact events, providing a methodological reference for real-time recognition of high-intensity contact in sports scenarios.

### 3.2 Action pattern recognition

Following the acquisition of purified signals, the system must automatically segment discrete motion phases (e.g., start acceleration, mid-run sprinting, and braking deceleration) from continuous electrical streams. Convolutional Neural Networks (CNNs), through their unique receptive field mechanisms, exhibit powerful advantages analogous to computer vision in extracting morphological waveform features (Alomar et al., 2025; Farha and Gall, 2019). Research indicates that multi-dimensional CNNs can map flexible sensing signals distributed across key lower-limb muscle groups into two-dimensional feature maps (Lea et al., 2017), thereby accurately capturing the “temporal fingerprints” of Ground Reaction Force (GRF) waveforms. The encoder-decoder Temporal Convolutional Network (TCN) first proposed by Lea et al. (2017) proved that CNNs outperform traditional recurrent neural networks in temporal modeling. Farha and Gall (2019) further introduced the Multi-Stage Temporal Convolutional Network (MS-TCN), which effectively mitigates the over-segmentation problem in long-sequence action segmentation by stacking multiple refinement stages. Building on this, the MSLID-TCN model introduced linear-index dilated convolutions to assign appropriate receptive fields to each layer, enabling the transfer of short- and long-term dependencies to subsequent layers (Gao et al., 2025). Kang et al. (2023) integrated multi-head attention mechanisms and segment-wise smoothing loss functions to achieve refined boundary recognition of action phases. Ferreira et al. 2022) validated the efficacy of DL methods in multi-phase segmentation tasks for fitness training. Such automated segmentation logic resolves the bottleneck of manual data labeling in sports science and provides standardized input tensors for subsequent phase-specific fatigue analysis.

### 3.3 Fatigue temporal feature mining

Motion-induced fatigue is essentially a dynamic cumulative process with high temporal dependency, manifesting as non-linear drifts in biomechanical characteristics over time (Wang and Ma, 2026). The Mamba-SportsNet framework suggests that the precise assessment of athletic performance via wearable sensor networks is currently constrained by the difficulty of modeling long-range dependencies. Since fatigue is a slowly evolving pattern, it requires model architectures capable of effectively processing long-sequence dependencies; traditional static recognition models fail to characterize these subtle evolutions across thousands of gait cycles.

Long Short-Term Memory (LSTM) networks and their variant, Gated Recurrent Units (GRU), have become core tools for monitoring fatigue evolution by utilizing forget-gate mechanisms to resolve the gradient vanishing problem inherent in traditional Recurrent Neural Networks (RNNs) (Li et al., 2024; Chua, 2025). An LSTM model based on wearable EMG data achieved a 99.65% classification accuracy in distinguishing between fatigued and non-fatigued states, validating its superior performance in muscle fatigue detection (Kannan et al., 2025). Li et al. systematically compared LSTM, GRU, and Transformer models in detecting abnormal running postures, confirming the effectiveness of LSTM/GRU in processing temporal motion signals, Chua utilized LSTM models to detect fatigue-induced muscle compensation behaviors, demonstrating that LSTM-based fusion of IMU and strain sensor data outperforms single-sensor solutions in capturing temporal fatigue characteristics (Li et al., 2024; Chua, 2025).

Latest research has introduced self-attention mechanisms, enabling models to automatically focus on mechanical failure points at the end of training sessions to predict the critical moment of Time-to-Exhaustion (TTE). A proposed CNN-LSTM-Attention hybrid model fused sEMG and IMU signals and achieved an 87.94% accuracy in fatigue state detection using leave-one-subject-out cross-validation, surpassing traditional subject-dependent models (Hwang et al., 2025). A TCN-RNN hybrid framework for cycling fatigue monitoring achieved an average prediction accuracy of 89.20%, significantly outperforming standalone LSTM, GRU, or Transformer architectures (Shen et al., 2025). Wu constructed an AI-driven model using big data and machine learning to identify key risk factors such as peak joint loads and muscle fatigue indices via LSTM networks, achieving an 89.7% injury prediction accuracy (AUC = 0.93), thereby providing decision support for preventing acute injuries caused by technical deformation (Wu, 2026).

### 3.4 Adaptation mechanisms between model architectures and biomechanical signals

The selection of deep learning models is not arbitrary but depends on the physical attributes of the signals generated by the underlying sensing hardware and the specific monitoring objectives, as detailed in Table 2. Building upon the hardware adaptation matrix established in Section 2.5 (Table 1), the raw biomechanical signals captured by various flexible sensors must be mapped onto corresponding mathematical representations to form a closed-loop “Physical Sensing to Digital Intelligence” pipeline.

**TABLE 2:** Algorithmic Adaptation Matrix of deep learning architectures in sports monitoring and injury early warning.

AI architecture	Adapted signal type	Core algorithmic advantage	Functional role in sports monitoring	Target fatigue/Injury scenario	Ref
CNN	morphological waveforms from piezoresistive/strain sensors	spatial feature extraction: receptive fields identify signal visual features	action phase segmentation: recognizes discrete phases like start, sprint, and brake	standardized technical assessment and normative scoring	Alomar et al, (2025), Lea et al. (2017)
LSTM/GRU	long-term, high-dimensional kinetic drift signals	temporal memory mechanism: forget gates and memory cells handle long-range dependencies	fatigue evolution mining: compares signal fingerprint deviations between early and late training	central fatigue identification; cadence and contact time variability	Li et al. (2024), Chua (2025)
Transformer	multi-node, multi-modal fused kinetic chain signals	self-attention: instantly locates anomalies within massive stride datasets	transient high-risk capture: identifies instability across multi-joint synergies from a global perspective	acute injury early warning (e.g., ACL risk); joint mechanical failure prediction	Wang and Ma (2026), Shen et al. (2025)
GNN	topological distributed sensor networks (e.g., ankle-knee-hip system)	non-Euclidean modeling: Abstracts human structure into point-line kinetic graphs	kinetic chain consistency: monitors force transfer efficiency and limb coordination	compensatory technical deformation; systemic kinetic chain dysfunction	Shao et al. (2024)

When utilizing piezoresistive or strain sensors to capture morphological waveforms of limb movements, the convolutional kernels of a CNN can identify signal shapes, making it suitable for automatic action phase segmentation and spatial classification tasks. Conversely, because fatigue is a dynamic drifting process, LSTM networks equipped with forget gates and memory cells can compare signal variances between an athlete’s initial and final training phases. Thus, in central fatigue recognition, LSTM serves as the core algorithm for characterizing cadence stability and contact time variability. In the context of multi-joint kinetic chain monitoring, Transformer models can instantly locate the most critical mechanical failure points within ten thousand strides. Their self-attention mechanisms provide significantly higher sensitivity for early warning of high-risk movements associated with acute injuries compared to traditional RNNs.

However, it is critical to acknowledge that while these deep learning architectures offer high predictive accuracy, their interpretability remains a challenge in practical sports monitoring. In high-stakes coaching and clinical practice, understanding the biomechanical rationale behind a prediction is as valuable as the prediction itself. Future systems should prioritize the integration of explainable AI (XAI) frameworks such as SHAP, lime, or attention-based relevance scores to bridge the gap between predictive accuracy and clinical interpretability, ensuring that model outputs are transparent and actionable for coaches and clinicians.

## 4 Biomechanical identification logic of exercise-induced fatigue

**FIGURE 2:**
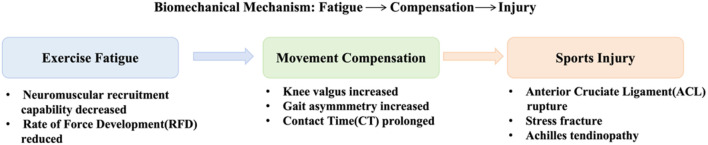
Biomechanical early warning logic model based on the “fatigue–compensation–injury“ causal chain.

The core of transducing flexible sensing signals into injury early warning models lies in establishing a biomechanical risk assessment paradigm based on the “fatigue–compensation–injury” causal chain. According to the injury etiology model proposed by Bahr and Krosshaug (2005), the occurrence of an injury depends on the imbalance between the mechanical load sustained by tissues and their structural load-bearing capacity, with neuromuscular fatigue serving as the critical trigger that disrupts this equilibrium (Windt and Gabbett, 2017). The dynamic evolutionary pathway from exercise-induced fatigue to tissue injury is illustrated in Figure 2 Flexible sensors facilitate early intervention by capturing biomechanical anomaly signals during the compensatory stage.

### 4.1 Real-time monitoring of acute sports injuries and high-risk movement identification

The core of early warning for acute injuries (e.g., Anterior Cruciate Ligament ruptures, severe ankle sprains) lies in the instantaneous capture of high-risk movement patterns. Due to their soft and conformal nature, electronic textiles (E-textiles) enable the integration of sensing functionalities into daily sportswear, facilitating continuous monitoring unrestricted by laboratory environments. Dong (2025) systematically investigated the fabrication and application of knit-based flexible sensors, developing various scalable sensors including triboelectric carpet fabrics, triple-layered plush sensing fabrics, and auxetic knitted strain yarns. The graphene-hybrid knitted sensor fabricated by Shao et al. (2024) maintained an excellent conductivity of 37 S/m while exhibiting superior flexibility and skin-modulus matching; when coupled with a CNN-LSTM-Attention mechanism, it achieved 100% recognition accuracy for knee joint movements.

Regarding dynamic knee stability, biomechanical research has established that excessive knee valgus moments, poor trunk control, quadriceps over-activation, and bilateral asymmetry are potential high-risk factors for ACL injuries. A systematic review by Belkhelladi et al. (encompassing 28 studies and 2,819 athletes) confirmed that trunk flexion/perturbation affects ACL loading risk in 83% of studies, hip abduction/internal rotation angles correlate with risk in 83%, and reduced knee flexion angles lead to increased ACL loading in 100% of the reviewed literature (Belkhelladi et al., 2025). Furthermore, Li L. et al. (2025) demonstrated that in complex, unanticipated environments, alterations in an athlete’s reactive movement patterns lead to abnormal landing biomechanics, further escalating the risk of ACL loading.

Tang et al. (2025) developed a hierarchical intelligent sensing platform integrating textile-based sEMG electrodes, ultra-sensitive textile strain sensors, and IMUs to achieve real-time perception across bone, muscle, and skin levels. The system achieved a root-mean-square error (RMSE) of 0.13 Nm/kg in ankle torque estimation, 97.1% accuracy in metabolic trend classification, and risk detection within 100 m with a recall rate of 0.96. This research validates the technical feasibility of fusing multi-modal flexible sensing with deep learning for acute injury early warning.

### 4.2 Cumulative load evaluation and asymmetry early warning for overuse injuries

In contrast to acute injuries, early warning for overuse injuries (e.g., stress fractures, Achilles tendinopathy) relies more heavily on the continuous monitoring of kinetic features within long-term time-series data. Biermaier et al. (2021) noted that while E-textiles have numerous applications in sports, safety, and healthcare, functional aging—including loss of sensing performance and degradation of conductivity—is critical to the reliability of long-term monitoring. This study attempted to establish cumulative fatigue injury theoretical models to simulate performance changes in sensing textiles over their lifecycle.

Gait asymmetry is regarded as a vital biomechanical manifestation of fatigue accumulation. In fact, most individuals possess functional asymmetries that influence force transmission pathways and alter systemic load distribution, serving as significant risk factors for overuse injuries. Research utilizing flexible plantar pressure-sensing textiles enables continuous monitoring of bilateral ground reaction force and impulse distributions. Plantar pressure measurement is widely used to diagnose lower-limb pathological states, as abnormal or excessive pressures often correlate with pain and injury risk. Chen et al. (2018) utilized a plantar pressure measurement system to record peak pressures and maximum force values in basketball athletes during various directional changes, confirming that different cutting maneuvers generate distinct plantar loading patterns; localized abnormal load elevation is closely associated with lower-limb overuse injury risk. Previously, bilateral plantar pressure asymmetry was validated as an effective indicator for identifying pedal pathological states (Sneyers et al., 1995).

Traditional injury prediction models often rely on external load metrics such as running distance; however, models based on flexible sensing textiles can evaluate internal load in real-time by estimating vertical ground reaction force (vGRF) signals. Indeed, textile-based sensors provide significant value in performance tracking, muscular measurement, and injury prevention. Etana et al. (2023) further emphasized that textile electrodes offer unique advantages for continuous physiological monitoring and performance assessment due to their suitability for long-term wear. By integrating the energy dissipation rate of each step over time, an athlete’s cumulative mechanical load profile can be constructed.

In the domain of deep learning-based fatigue recognition, recent research (Hwang et al., 2025) employed a hybrid CNN-LSTM-Attention model to process multi-modal signals from sEMG and IMUs. In a study of 35 healthy subjects, the model achieved a detection accuracy of 87.94% and a balanced recall of 87.94% using leave-one-subject-out cross-validation. These results validate the efficacy of DL models in mining temporal fatigue features and provide algorithmic support for constructing cumulative load evaluation systems based on flexible sensing textiles.

In summary, the decline in motor function does not occur in isolation but follows a pathophysiological pathway from physiological fatigue to biomechanical compensation, eventually culminating in tissue injury. To provide a more intuitive representation of the mapping between fatigue, compensatory behavior, and flexible sensing signals during this evolution, Table 3 summarizes the risk early warning classification matrix based on the “fatigue–compensation–injury” causal chain.

**TABLE 3:** Risk early warning classification matrix based on the “fatigue–compensation–injury” causal chain.

Injury risk category	Associated fatigue type	Key biomechanical compensatory features	Flexible sensing fingerprints (signal expression)	Potential sports injury risks
Acute Injury	instantaneous decline in neuromuscular function	increased knee valgus; increased landing stiffness	rightward shift of strain peak; steep increase in voltage gradient	ACL rupture; severe ankle sprain
Overuse Injury	cumulative fatigue of the kinetic chain	bilateral gait asymmetry >15%	significant divergence in bilateral sensor voltage impulse	stress fracture; Achilles tendinopathy; Iliotibial band syndrome
Technical Regression	fatigue of fine motor control	restricted joint ROM; decreased movement consistency	reduced signal autocorrelation; severe distortion of waveform envelope	muscle strain; inefficient training due to technical deformation

As shown in Table 3, flexible sensing textiles, by virtue of their high sensitivity and sampling frequency, can capture microscopic mechanical compensations unrecognizable to the naked eye or traditional video analysis, transducing them into quantifiable electrical fingerprints. Crucially, these quantitative sensing fingerprints are precisely designed to serve as the target patterns that the deep learning architectures reviewed in Section 3 automatically learn to recognize and classify. By positioning the deep learning networks as the non-linear computational engine and these fingerprints as the biomechanical targets, this alignment establishes a complete, end-to-end closed-loop framework for intelligent risk assessment without relying on restrictive classical equations. This provides a precise logical trigger for the intelligent early warning and real-time feedback loops described in Section 4.3.

### 4.3 Biomechanical feedback and intelligent decision-making loops

The ultimate objective of injury early warning is to facilitate effective intervention. Current research is driving the evolution of smart sports apparel from simple sensor terminals to closed-loop intervention systems.

The primary prerequisite for a closed-loop system is the ability of sensors to operate stably over long durations without being constrained by battery life. Advances in Triboelectric Nanogenerator technology provide a new pathway for self-powered sensing. Wang Q. et al. (2025) developed a fully integrated, self-powered, wireless smart insole for gait monitoring and real-time analysis. This insole integrates 22 pressure sensors and utilizes a nonlinear synergistic strategy to achieve superior linearity (R^2^ > 0.999, pressure range 0–225 kPa) and high durability (>180,000 cycles), with power provided by solar cells. Combined with a Support Vector Machine (SVM) model, the system accurately recognizes eight motion states, including sitting, standing, and running. Lu et al. (2023) developed a lightweight, portable, self-powered TENG system to monitor the stroke frequency and force of ice skaters, assisting coaches in refining training methods via wireless data transmission and visualized feedback. These studies demonstrate that self-powered technologies provide the hardware foundation for the long-term deployment of closed-loop systems.

Following the acquisition of continuous monitoring data, the effective extraction of features relevant to fatigue and injury risk from massive, high-dimensional biological signals remains a critical challenge. Recent research in biosignal time-series analysis indicates that while wearable sensors enable continuous health monitoring, the scarcity of labeled data is a core obstacle. A proposed contrastive self-supervised learning framework enhances label efficiency by learning effective representations from unlabeled data, while foundational models for biosignals improve generalization across tasks and datasets (Wu, 2025). Utilizing transfer learning and semi-supervised frameworks, systems can dynamically adjust early warning sensitivity based on an athlete’s injury history, strength baselines, and current fatigue indices, achieving individualized monitoring. Indeed, research on flexible wearable medical devices underscores the pivotal role of AI and machine learning in processing complex physiological data for predictive diagnostics and personalized health management (Zhou et al., 2026).

Based on the aforementioned self-powered hardware and individualized modeling, the final output of the closed-loop system is intelligent intervention decision-making. Machine learning-driven wearable systems can achieve adaptive load control and subclinical injury detection, promoting a paradigm shift in injury prevention from empirical intervention to intelligent prediction. By constructing a “dynamic perception–intelligent analysis–autonomous regulation” closed-loop system, the athletic protection architecture is evolving from population-wide generalizations to individualized precision. This represents the ultimate application value of flexible sensing textiles in smart sports.

## 5 Injury risk assessment models and intelligent decision-making pathways

### 5.1 Cumulative load quantification and risk threshold modeling

Compared to acute injuries, the early warning of overuse injuries (e.g., stress fractures, Achilles tendinopathy) relies more heavily on the continuous monitoring of kinetic features in long-term time-series data and the precise quantification of cumulative load.

As a foundational baseline establishing traditional rigid hardware and statistical formulations, Weiss et al. (2017), through a season-long longitudinal study of professional basketball players using wearable accelerometers to monitor impact frequencies at varying intensities, found that injury incidence was lowest (36%) when the acute-to-chronic workload ratio (ACWR) was maintained between 1.0 and 1.5. Conversely, when the ACWR was ≤0.5 or ≥1.5, injury rates escalated to 54% and 59%, respectively. While such deterministic statistical benchmarks provide valuable macroscopic boundaries, they lack the capacity to adaptively decode complex physiological signals. Transitioning from these conventional rigid-device metrics, the integration of advanced flexible sensing textiles with data-driven deep learning models provides a more ergonomic, comprehensive, and predictive landscape of the workload-injury relationship.

In the application of digital twin technology, Sharotry et al. (2022) proposed a biomechanical fatigue detection framework based on digital twin modeling. This framework detects fatigue by analyzing variations in back, elbow, and knee joint angles during manual material handling tasks. It employs Dynamic Time Warping (DTW) to analyze temporal variations in joint angles and uses Exponentially Weighted Moving Average (EWMA) control charts to evaluate shifts in fatigue parameters. Their research indicates that different individual’s manifest fatigue through distinct joints, highlighting the necessity of personalized digital twin models. Building on this, subsequent research has integrated digital twins with wearable sensing (IMU, EMG) and machine learning to capture user movement in real-time, comparing it against predefined biomechanical thresholds to predict fatigue and injury risks. Feedback is then delivered via video, audio, or haptic alerts (Sharotry et al., 2025).

### 5.2 Individualized monitoring and digital twin-driven early warning benchmarks

The core challenge in injury early warning is the inherent inter-individual heterogeneity of athletes, where uniform mechanical thresholds often result in high rates of false positives or missed detections. Moving beyond universal injury traits, the Personalized Athlete Injury Risk (PAIR) model proposed by Ozolcer and Bae (2025) utilizes individualized embeddings to capture athlete-specific patterns. Evaluated on over 3,000 daily observations from 36 collegiate athletes, the model achieved an R^2^ of 0.506, significantly outperforming the non-personalized baseline of 0.302.

While this improvement is statistically and practically meaningful, its utility must be interpreted cautiously within the stochastic context of field-based athletics. Given the multifactorial etiology of injury, this R^2^ value should function as a decision-support indicator rather than a deterministic diagnostic tool. To mitigate overfitting, the model was rigorously evaluated using leave-one-subject-out (LOSO) cross-validation a protocol that tests performance on unseen individuals to confirm that performance gains reflect genuine individualization rather than spurious correlations. Nevertheless, future robustness can be further enhanced through regularization techniques such as Dropout or L2-norm penalties. Finally, as the current framework was trained exclusively on collegiate data, its generalizability remains a limitation. Consequently, future research should prioritize transfer learning and multi-center external validation to expand its clinical utility across diverse populations, such as elite professionals or adolescent athletes. Through such personalized frameworks and multimodal data fusion, injury early warning systems can achieve comprehensive, low-latency solutions from data acquisition to edge-based decision-making.

### 5.3 Ecological validity and intelligent decision pathways

As illustrated by the empirical studies summarized in Table 4 (which delineates the paradigm shift from traditional statistical and classical kinematic baselines to modern deep learning implementations), the field is currently transitioning from simple signal acquisition toward complex risk prediction based on digital twins, textile-based intelligence, and personalized models.

**TABLE 4:** Summary of representative applications of flexible sensing textiles fused with AI in sports monitoring.

Representative study (Year)	Monitoring objective	Sensor technology path	AI/Algorithmic architecture	Key outcomes & performance	Ref
Shao et al. (2024)	complex knee motion recognition	graphene-hybrid knitted fabric	CNN-LSTM-Attention	100% recognition accuracy for multi-dimensional knee movements	Shao et al. (2024)
Tang et al. (2025)	subclinical injury risk detection	textile sEMG and Strain multimodal	hierarchical intelligent sensing platform	risk detection latency <100 m; recall rate of 0.96	Tang et al. (2025)
Ozolcer et al. (2025)	individualized injury risk prediction	wearable multi-source big data	personalized athlete injury risk	R^2^ = 0.506, significant improvement via individualized embeddings	Ozolcer and Bae (2025)
Weiss et al. (2017)	seasonal cumulative load evaluation	accelerometer and kinetic sensing	Statistical ACWR formula (Non-DL baseline)	identified the 1.0–1.5 sweet spot to minimize injury rates	Weiss et al. (2017)
Fan et al. (2021)	*in-situ* ACL risk assessment	IMU and flexible sensing hybrid	Kinematic dynamic estimation (Classical algorithm)	knee valgus error <3°; high-precision non-lab monitoring	Fan et al. (2021)

Despite the significant technical breakthroughs shown in Table 4, bridging the ecological gap remains a prerequisite for the transition from laboratory prototypes to field-based athletic practice. Current AI models are largely trained on standardized movement libraries; however, unanticipated changes in direction, high-intensity collisions, and complex field conditions in real competition often led to significant biomechanical drift. This risks the accuracy of models validated only in laboratory settings. Consequently, the academic community has begun to deeply investigate the discrepancies between indoor and outdoor athletic performance to construct more robust early warning benchmarks through *in-situ* data calibration.

Laboratory-based research is often constrained by a lack of ecological validity. Di Paolo et al. (2023) compared the agility kinematics of elite youth female football players in laboratory versus on-field environments, finding significant kinematic discrepancies. On-field conditions exhibited a lower sagittal plane range of motion, and greater ankle valgus and pelvic rotation (p < 0.044), with the most pronounced differences occurring during the landing and weight-bearing phases. This study suggests that athletes should be tested in ecologically valid environments to refine ACL injury prevention protocols. Representing a high-precision classical kinematic benchmark, research by Fan et al. (2021) confirmed that deterministic IMU-based knee angle estimation algorithms can achieve root-mean-square errors (RMSE) of 1.07°, 2.87°, and 2.64° for knee flexion, abduction, and internal rotation during landing and cutting tasks. While this provides a robust mathematical foundation for wearable ACL risk assessment outside the laboratory using conventional rigid hardware, such classical kinematic algorithms are primarily tailored for linear sensor inputs. To decode the highly non-linear and hysteretic signals inherent in advanced flexible sensing textiles, transitioning from these deterministic algorithms to data-driven deep learning models becomes essential.

To maintain cross-scenario consistency outside the laboratory, researchers are increasingly adopting *in-situ* calibration strategies and adaptive data fusion schemes. One promising approach is the implementation of transfer learning-based domain adaptation, where deep learning models pre-trained on laboratory-standardized movements are fine-tuned using small-batch, unlabeled field data to mitigate environmental distribution shifts. Furthermore, for sensor signals prone to baseline drift due to skin-textile interface changes, dynamic reference normalization, a process that periodically calibrates the sensor’s zero-point against known ‘static’ movement phases has proven effective in ensuring temporal consistency. Additionally, multimodal data fusion, such as integrating high-frequency IMU data with low-frequency strain signals, enables the system to cross-verify biomechanical metrics. Specifically, if a specific strain signal drifts due to sensor displacement, the algorithm cross-references IMU-derived limb orientation to compensate for the error in real-time.

Building upon these individualized monitoring benchmarks and field-ready risk assessment methods, injury early warning systems ultimately facilitate intelligent intervention decisions. By constructing a “dynamic perception–intelligent analysis–autonomous regulation” closed-loop system, the athletic protection paradigm is evolving from population-wide generalizations to individualized precision. This represents the ultimate application value of fusing flexible sensing textiles with deep learning in smart sports and serves as the cornerstone for the new paradigm of digital athletics.

## 6 Challenges and future perspectives

Despite the exceptional capabilities demonstrated by the fusion of flexible sensing textiles and deep learning for fatigue identification and injury early warning in laboratory settings, a comprehensive transition from prototype development to field-based athletic practice remains contingent upon overcoming a series of critical challenges across the physical, engineering, and systemic domains.

### 6.1 Hardware-level reliability and standardized durability benchmarks

The primary challenges for textile-based sensing systems in practical applications include mechanical degradation post-laundry, electrode delamination, and scalability constraints. Research indicates that mechanical stress and chemical erosion during washing cycles lead to the loss of active nanomaterials and a decrease in interfacial bonding strength. Incorporating nanomaterials as anchoring points has been shown to effectively enhance interfacial adhesion, thereby improving washability. To address these stability issues, researchers are developing comprehensive encapsulation strategies, including superhydrophobic coatings and *in-situ* lamination techniques. Recent studies utilizing an alternating-layer structure of reduced graphene oxide (rGO) and polyaniline (PANI) have produced electronic textiles that maintain excellent sensing durability achieving a Gauge Factor (GF) of 39.7 and a strain range of 0.0625%–200% even after 1,500 cycles of stretching, bending, twisting, and rigorous machine washing, while retaining breathability superior to pure cotton (Sun et al., 2024). Furthermore, electronic yarn (E-yarn) technology, which embeds electronic components within flexible yarn structures encapsulated in polymer resins, has demonstrated favorable durability across various integration methods (Shahidi et al., 2025).

However, the current lack of standardized wash-test protocols hinders the direct comparison of results across different studies, especially considering that the laundry frequency of elite athletes far exceeds the testing ranges of existing literature (Rotzler and Schneider-Ramelow, 2022). To bridge the gap between empirical laboratory prototypes and field deployment, emerging research must transition from arbitrary home-brewed testing to rigorous international engineering standards. For instance, textile electronics must be benchmarked against AATCC TS1 for electronic textile washability and ISO 6330 for domestic laundering procedures, defining a post-wash Signal-to-Noise Ratio (SNR) retention (e.g., $>20$ dB) after 30+ standardized cycles as a baseline reliability threshold. The integration of machine learning-aided design and embedded energy storage systems holds promise for further enhancing the long-term stability of textile-based sensors.

### 6.2 Signal fidelity under extreme hyperhidrosis and high-impact motion artifacts

During high-intensity scenarios such as football tackling or sprinting, relative slip between the fabric and skin induces severe motion artifacts, the magnitude of which often exceeds the biometric signals of interest. Research on dynamic soft tissue artifacts under impulsive loads has revealed that wearable inertial measurement units can overestimate peak vertical acceleration by 1.55–2.18 times; while low-pass filtering can mitigate this overestimation, it simultaneously attenuates the reference signal (Rong and Kuo, 2024). Furthermore, the perspiration generated during intense exercise not only alters the dielectric constant of the textile but also shields surface charges in triboelectric sensors, leading to significant signal distortion.

Under these real-world coupled stressors—hyperhidrosis and severe mechanical impacts—empirical evidence for long-term signal stability remains scarce. To address this, devices must be subjected to structural and chemical stressors regulated by industrial standards, such as ASTM D5276 for high-impact ruggedness and ISO 105-E04 for artificial sweat corrosion resistance. Quantitatively, a field-ready sensor should ideally maintain a baseline electrical drift $\left(\Delta R/R_{0}\;or\;\Delta V/V_{0}\right)$ of <5% and a Gauge Factor attenuation $\left(\Delta \text{GF}/GF_{0}\right)$ within a negligible threshold after surviving at least 10,000 continuous cyclic deformations under a synthetic perspiration environment. Furthermore, ensuring a stable Water Vapor Transmission Rate (WVTR) exceeding 2000 g/m^2^ 24 h is essential to minimize sweat accumulation and sweat-induced sensor slippage. To resolve the unreliability of single-modality signals in dynamic environments, researchers have proposed multimodal fusion strategies. By utilizing inertia-guided reliability modulation mechanisms, kinematic data can be used to dynamically weigh physiological signals, effectively suppressing the interference of motion artifacts on physiological data.

### 6.3 Systemic evolution: from solitary sensing to multimodal wearable ai coaches

The ultimate evolution of intelligent sports monitoring will transcend solitary mechanical sensing, moving toward a “Force–Physiology–Environment” multimodal fusion. Integrating flexible strain sensors with textile electrodes allows for the simultaneous acquisition of an athlete’s external and internal loads. Such fused sensing frameworks can consolidate signals from ECG, photoplethysmography, and IMUs to achieve unified human activity recognition, biometric identification, and health analytics. In the context of multimodal fusion, physiology-enhanced attention mechanisms can leverage physiological signals to provide context for motion patterns, enhancing the system’s ability to precisely locate and classify complex activities within long-duration sequences. Cross-modal fusion frameworks, which jointly learn sensing timing and inference methods, maintain stable performance even under sensor gating or missing input conditions, while significantly reducing sensor utilization and improving accuracy.

By incorporating environmental sensor data, systems will be capable of differentiating between states that are physiologically similar but etiologically distinct, such as exercise-induced tachycardia versus stress-related palpitations. Combined with cloud-based big data and personalized models, this will facilitate the construction of a human digital twin for every athlete. Future smart textiles will not only provide early warnings but will also offer instantaneous action guidance through integrated haptic feedback units. This evolution from simple sensors to wearable AI coaches will fundamentally reshape the training and protection paradigms of competitive sports.

## 7 Conclusion

The synergy between flexible sensing textiles and deep learning is fundamentally reshaping the landscape of athletic biomechanical monitoring. This review systematically demonstrates that the transition from constrained laboratory settings to ecologically valid field environments is no longer a technological aspiration but a nascent reality. Through the integration of advanced materials such as triboelectric nanogenerators, MXenes, and liquid metals, flexible hardware now provides a high-fidelity, unobtrusive medium for capturing the subtle kinetic and kinematic fingerprints of movement. Simultaneously, the evolution of deep learning architectures transitioning from spatial feature extraction (CNN) to temporal dependency modeling (LSTM) and global attention mechanisms (Transformers) facilitates the accurate decoding of these complex signals into actionable physiological insights.

Crucially, this paper establishes a unified theoretical framework based on the “fatigue–compensation–injury” causal chain. By identifying biomechanical drift during the compensatory stage, such as gait asymmetry and kinetic chain instability, the proposed system enables the preemptive interception of injury risks before clinical symptoms manifest. However, realizing the full potential of these advancements requires overcoming significant methodological and engineering barriers. First, to transition from predictive accuracy to clinical trust, future research must prioritize the integration of Explainable AI (XAI) frameworks and Physics-Informed Neural Networks (PINNs), ensuring that model outputs are grounded in biomechanical causality rather than opaque “black-box” correlations. Second, moving toward the vision of human digital twins and wearable AI coaches necessitates overcoming three core bottlenecks: (1) hardware-level, energy-efficient edge computing for real-time, low-latency kinematic streaming; (2) data-level standardization to unify heterogeneous signals across diverse sensor architectures; and (3) algorithmic breakthroughs in hybrid modeling, specifically fusing data-driven deep learning with physics-based musculoskeletal constraints to ensure the digital twin behaves with biological fidelity.

While persistent challenges regarding sensor washability, signal artifacts, and individualized model generalization remain, the trajectory toward integrated wearable AI is clear. In conclusion, the profound fusion of flexible electronics and artificial intelligence serves as the cornerstone for a new paradigm of evidence-based, individualized, and proactive sports medicine, effectively bridging the gap between high-tech monitoring and tangible athletic protection.
